# Potential Antifungal Targets against a Candida Biofilm Based on an Enzyme in the Arachidonic Acid Cascade—A Review

**DOI:** 10.3389/fmicb.2016.01925

**Published:** 2016-12-06

**Authors:** Xinning Liu, Decai Wang, Cuixiang Yu, Tao Li, Jianqiao Liu, Shujuan Sun

**Affiliations:** ^1^Department of Clinical Pharmacy, Taishan Medical UniversityTaian, China; ^2^Respiration Medicine, Qianfoshan Hospital Affiliated to Shandong UniversityJinan, China; ^3^Intensive Care Unit, Qianfoshan Hospital Affiliated to Shandong UniversityJinnan, China; ^4^General Practice, Shandong Provincial HospitalJinnan, China; ^5^Pharmaceutical Department, Qianfoshan Hospital Affiliated to Shandong UniversityJinnan, China

**Keywords:** *Candida albicans*, antifungal, arachidonic acid cascade, prostaglandin E2, mPGES-1

## Abstract

Candida is an important opportunistic fungal pathogen, especially in biofilm associated infections. The formation of a Candida biofilm can decrease Candida sensitivity to antifungal drugs and cause drug resistance. Although many effective antifungal drugs are available, their applications are limited due to their high toxicity and cost. Seeking new antifungal agents that are effective against biofilm-associated infection is an urgent need. Many research efforts are underway, and some progress has been made in this field. It has been shown that the arachidonic acid cascade plays an important role in fungal morphogenesis and pathogenicity. Notably, prostaglandin E2 (PGE_2_) can promote the formation of a Candida biofilm. Recently, the inhibition of PGE_2_ has received much attention. Studies have shown that cyclooxygenase (COX) inhibitors, such as aspirin, ibuprofen, and indomethacin, combined with fluconazole can significantly reduce Candida adhesion and biofilm development and increase fluconazole susceptibility; the MIC of fluconazole can be decrease from 64 to 2 μg/ml when used in combination with ibuprofen. In addition, *in vivo* studies have also confirmed the antifungal activities of these inhibitors. In this article, we mainly review the relationship between PGE_2_ and Candida biofilm, summarize the antifungal activities of COX inhibitors and analyze the possible antifungal activity of microsomal prostaglandin E synthase-1 (MPGES-1) inhibitors; additionally, other factors that influence PGE_2_ production are also discussed. Hopefully this review can disclose potential antifungal targets based on the arachidonic acid cascade and provide a prevailing strategy to alleviate *Candida albicans* biofilm formation.

## Introduction

*Candida albicans*, as a commensal microorganism of the human microbiome and a type of major fungal pathogen, is capable of causing disseminated or chronic infections. These infections often come with high mortality and morbidity in critically ill patients and immunocompromised individuals, such as AIDS patients, patients undergoing anticancer therapies, and so on (Carrillo-Muñoz et al., 2006). The National Healthcare Safety Network (NHSN) at the Centers of Diseases Control and Prevention (CDC) has reported that *Candida* spp. ranked fifth among hospital-acquired pathogens (Sievert et al., 2013). Fluconazole is one of the most commonly used antifungal drugs for human candidiasis; however, its extensive use has increased Candida resistance and led to refractory fungal infection (Silva et al., 2012). In addition, *C. albicans* can easily form a biofilm on the surface of catheters and other medical devices, which is the main cause of biomaterial-related infections. The National Institutes of Health reported that biofilms are responsible for over 80% of all microbial infections in the United States (Fox and Nobile, 2012). Therefore, identifying efficacious drugs that inhibit biofilm formation is critical to overcome the resistance of *C. albicans*. The arachidonic acid (AA) cascade is essential for mediating human biological activity and plays an important role in fungal morphogenesis and growth. Almost every key enzyme of the AA cascade has been the subject of pharmaceutical research (Meirer et al., 2014). AA is the main precursor of eicosanoids, and AA can be released from membrane phospholipids after catalysis by phospholipases (Dennis et al., 2011) and can be metabolized into many types of biologically active eicosanoids via the action of three separate groups of enzymes: cyclooxygenases, lipoxygenases (LOX), and cytochrome P450 (CYP). Lipoxygenase and cytochrome P450 catalyzes leukotrienes (LT; Peters-Golden and Henderson, 2007) and epoxyeicosatrienoic acids (EET; Spector, 2009), respectively. As the most popular enzyme, COX catalyzes the formation of thromboxane (TX), and prostaglandins (PG; Clària, 2003). PGE_2_, the most abundant PG, has been previously reported to mediate several biological phenomena, such as homeostasis (Sugimoto et al., 2000), inflammation, pain (Davies et al., 1984), and tumorigenesis (Wang and Dubois, 2010). Recent research has revealed that PGE_2_ is produced by human eukaryotic cells as well as by pathogenic fungi (Noverr et al., 2001, 2002). AA in combination with antifungal agents can affect PGE_2_ levels in several Candida species (Mishra et al., 2014). Some studies have suggested that fungal prostaglandin can act as a regulator for biofilm development in *C. albicans* and that it is also a significant virulence factor in biofilm-associated infections of *C. albicans* (Alem and Douglas, 2005). In this review, we mainly discussed the role of PGE_2_ in mediating *C. albicans* biofilm formation and the antifungal activity of the COX inhibitor of the arachidonic acid cascade, as well as other impact factors that influence the formation of PGE_2_.

## PGE_2_ and candida biofilm

The pathogenicity of *C. albicans* includes several virulence factors, such as adhesion, biofilm formation, and phenotypic switching (Calderone and Fonzi, 2001). The proclivity of *C. albicans* to form biofilms has caused a range of superficial mucosal infections and severe disseminated candidiasis (Fox and Nobile, 2012). A variety of urinary and central venous catheters are susceptible to *C. albicans* biofilm formation, and almost 50% of these catheters develop a biofilm infection (Nobile and Johnson, 2015). The treatment of catheter-related infections in the clinical setting is a challenge because *C. albicans* biofilm is intrinsically resistant to the host immune system and conventional antifungal drugs (Blankenship and Mitchell, 2006; Nobile and Johnson, 2015). The resistance of *C. albicans* biofilm cells to antifungal drugs is higher than that of planktonic cells, and the corresponding MICs were 30–2000 times higher (Douglas, 2003). Therefore, inhibiting biofilm formation is important for fungal resistance reversing. Recent researches show that PGE_2_ is able to regulate a diversity of host immune responses. It can inhibit Th1-type and promote Th2-type immune responses, which are responsible for regulating diverse homeostatic and inflammatory processes (Shibata et al., 2005). And imbalance of the Th response will cause chronic or disseminating fungal infections (Romani and Kaufmann, 1998). *C. albicans* has been reported to produce PGE_2_ in HeLa cells (Deva et al., 2001). Previous study certified that in mammalian cells, fungal PGE_2_ is able to down-modulate the production of chemokine and TNF-α, it exhibits the similar activities as mammalian PGE_2_, and that both are able to enhance fungal cell adhesion, biofilm development, and germ tube formation in *C. albicans* (Noverr et al., 2001). PGE_2_, as the regulator of the dimorphic structure of *C. albicans*, shows ability to increase intracellular cyclic AMP (cAMP) levels and stimulates *C. albicans* germ tube formation (Kalo-Klein and Witkin, 1990; Douglas, 2003). So enhancing PGE_2_ level during fungal infections can aggravate fungal colonization in biofilm formation and trigger chronic infection (Noverr et al., 2001). Evidence reveals that candidiasis is associated with high levels of PGE_2_ (Noverr et al., 2003), and decreased prostaglandin production during *C. albican* infections is an important factor in relieving chronic infections (Mishra et al., 2014). In addition, research suggests that PGE_2_ is produced in both *C. albicans* planktonic and biofilm cells (Ells et al., 2011), while biofilm cells secreted significantly more PGE_2_than the planktonic cells when determined according to cell dry weight. This may be one of the mechanisms that explain the high resistance of biofilm cells (Alem and Douglas, 2005). Further study showed that several genes play a role in regulating prostaglandin production in *C. albicans*. Levitin's work suggests that PGE_2_ can activate the signal transduction pathway of *C. albicans* by changing its transcriptional profile under yeast growth conditions, it indirectly down-regulate the expression of *C. albicans* homolog of *Ctr1* by activating *Tup1p* repressor and possibly other transcription factors (Levitin and Whiteway, 2007). According to Erb-Downward's research, fatty acid desaturase homolog (Ole2p) and multicopper oxidase homolog (Fet3p) are necessary enzymes that participate in PGE_2_ biosynthesis in *C. albicans* (Erb-Downward and Noverr, 2007), while the role of *OLE2* in *C. parapsilosis* is relatively weaker, it is able to decrease *C. parapsilosis* virulence, but is dispensable for PGE_2_ synthesis (Grozer et al., 2015). Furthermore, PGE_2_ production is a significant virulence factor in biofilm-associated infections of *C. albicans* and non-albicans species (Mishra et al., 2014), the relative lower virulence of *C. dubliniensis* compared to *C. albicans* may be contributed to its lower PGE_2_ level (Ells et al., 2011). The role of PGE_2_ in polymicrobial infection has also been studied. In mixed infections containing Candida and bacterial species, such as dual *S. aureus/C. albicans* polymicrobial biofilms and *C. albicans* in mixed infections with *Pseudomonas aeruginosa*, both of the pathogens have a tendency to produce PGE_2_ (Krause et al., 2015; Fourie et al., 2016) and cause nosocomial infections. PGE_2_, as the key molecule, could stimulate dual biofilm formation, and affect the dynamics of co-infection.

Above all, although there are multiple speculations of the role of PGE_2_ in *C. albicans* biofilm development and fungal pathogenesis, however, evidence shows that PGE_2_ has significant effects on fungal morphogenesis and biofilm development, It is indeed, in response to *C. albicans* infections, both host and fungal cells are sources of prostaglandins production and the host-derived arachidonic acid may be taken up by fungi for prostaglandin synthesis. On the other hand, the detail mechanism of PGE_2_ regulating biofilm formation and pathogenicity of fungi is not completely clear. So far, some research has shown that several transcriptional regulators control biofilm development in *C. albicans*. Among these regulators, the *CPH1* and *EFG1* genes are required for numerous processes, such as morphogenesis and virulence (García-Sánchez et al., 2004); *BCR1* is a downstream gene of the hyphal regulatory network that regulates biofilm formation, and *HWP1* is used for biofilm adherence in *C. albicans* (Li et al., 2015; Nobile and Johnson, 2015). However, whether PGE_2_ modulates biofilm formation of *C. albican*s by regulating the expression of these genes is still awaiting support from further experiments.

## Cyclooxygenase

The biosynthesis of PG is catalyzed by several enzymes. First, AA is mediated by COX-1 or COX-2 to form prostaglandin G2 (PGG_2_), and then, the same enzyme catalyzes the formation of prostaglandin H2 (PGH_2_). Finally, PGE_2_ is enzymatically produced as an end product of the reaction of PGE_2_ synthase (PGES; Park et al., 2006; Iyer et al., 2009). COX inhibitors include non-steroidal anti-Inflammatory drugs (NSAIDs), such as aspirin, indomethacin, ibuprofen, sodium salicylate, and diclofenac sodium, and these are the most widely used drugs in anti-inflammatory therapy. They are used to treat pain and inflammation in a variety of acute and chronic conditions by inhibiting both COX-1 and/or COX-2. PGE_2_ is a virulence factor in promoting fungal colonization and chronic infection, using pharmacologic agents which can reduce PGE_2_ production are therapeutic options for Candida related infection (Nash et al., 2016). It has been reported that in human urothelium, *C. albicans* can induce COX-2/PGE_2_ gene expression through EGFR-ERK/p38-RSK-CREB-1 pathway (Wang et al., 2016). Notably, recent studies have suggested that COX inhibitors had strong antifungal activity against *C. albicans* by a PGE_2_-dependent mechanism. It can inhibit fungal prostaglandin synthesis and therefore reduce biofilm development in *C. albicans* (Alem and Douglas, 2004, 2005; Ghalehnoo et al., 2010; Bink et al., 2012; Rusu et al., 2014). They inhibit the growth of *C. albicans* in a dose-dependent manner (de Quadros et al., 2011). Among them, indomethacin, a potent COX inhibitor, is most effective against *C. albicans* (MFC 0.11 mmol/L), followed by aspirin (MFC 0.22 mmol/L) and ibuprofen (MFC 0.44 mmol/L), while sodium salicylate isconsiderably less potent (MFC 22 mmol/L) than the other tested NSAIDs.

Research has shown that aspirin, one of the oldest and most widely used COX inhibitors, exerts desirable inhibitory effects on growing and mature biofilms (48 h) of *C. albicans* by inhibiting PGE_2_ synthesis (Alem and Douglas, 2004). It can significantly reduce biofilm synthesis in fluconazole resistant *C. albicans* clinical isolates at a concentration of 1 mg/ml and eliminate biofilm formation at a concentration of 5 mg/ml (Abdelmegeed and Shaaban, 2013). Aspirin also has significant effects on the viability of *C. albicans* biofilm cells. Aspirin-treated cells are incapable of cell division, but still retain some level of metabolic activity (Alem and Douglas, 2004). Currently, combination therapy has become a potential alternative treatment for invasive fungal infections, as it can exhibit improved efficacy, a broader spectrum of activity and fewer side effects (Shrestha et al., 2015). In other experiments, the antifungal ability of amphotericin B combined with aspirin against planktonic cells and biofilm cells of *C. albicans* has been evaluated. The antifungal activity of aspirin is weak in both planktonic and biofilm cells, but when aspirin is combined with amphotericin B, it enhances the antifungal activity of amphotericin B, especially on the biofilm cells. The MIC50 values of amphotericin B and aspirin are, respectively, decreased by up to 32- and 16-fold, based on FIC indices (Zhou et al., 2012). Aspirin alone or in combination with conventional antifungal drugs is also beneficial for the treatment of vulvovaginal candidiasis (Deva et al., 2001). In Ghalehnoo's study, diclofenac sodium was shown to have a strong inhibitory effect on filamentation in *C. albicans*. To clarify the ability of diclofenac sodium to reduce germ tubes and hyphal formation, a RT-qPCR experiment was used to observe the interference of gene expressions in *C. albicans*. The results show that the expression of *CYR1, EFG1*, and *RAS1* in the CAMP-EFG1 pathway were repressed by the presence of diclofenac sodium, while *CST20* and *CPH1* in the MAPK pathway were not, suggesting that diclofenac sodium may be involved in the yeast-hypha transition in *C. albicans* by disrupting the cAMP-EFG1 pathway (Ghalehnoo et al., 2010). Another study found that combining diclofenac with caspofungin led to a successful reduction of biofilm cells *in vitro* and *in vivo* and that the presence of diclofenac enhanced the susceptibility to caspofungin. In a catheter-associated biofilm model in rats, the combination of diclofenac with caspofungin treatment resulted in a > 15-fold significant reduction in biofilm cells compared to the control treatment, which showed a > 5-fold reduction (Bink et al., 2012). Yucesoy's experiment suggested that a combination of diclofenac sodium with fluconazole *in vitro* had a synergistic activity against fluconazole-resistant *C. albicans* strains (Yucesoy et al., 2000; Rainsford, 2007) and the MIC of fluconazole was decreased four-fold when combined with diclofenac sodium. These results are parallel with experiments on clinical isolates of *C. albicans* from AIDS patients (Scott et al., 1995). Non-steroidal anti-inflammatory drug flufenamic acid (FFA) alone or in combination with amphotericin B, caspofungin and fluconazole might be effective for the prevention of *C. albicans* biofilms (Chavez-Dozal et al., 2014). Ibuprofen, a NSAID, is crucial for the reversion of azole resistance in *C. albicans*. In a murine model of systemic infection, the combination of ibuprofen, and fluconazole can potentiate the antifungal activity of fluconazole by decreasing the MIC of fluconazole from 64 to 2 μg/ml and reduce the fungal burden and morbidity in fluconazole resistant strains (Pina-Vaz et al., 2000; Arai et al., 2005; Costa-de-Oliveira et al., 2015). As a potential Cdrp blocker, ibuprofen can directly damage *C. albicans* cell membranes, but the molecular mechanisms of ibuprofen against *C. albicans* are still being uncovered (Ricardo et al., 2009; de Quadros et al., 2011). Anidulafungin (ANF) is an antifungal drug that can inhibit the formation of both planktonic and biofilms cells in *C. albicans*. *In vitro*, when aspirin, ibuprofen, and diclofenac are combined with ANF, they show different sensitivity regarding decreasing biofilm formation in several *Candida* spp. The combination of ANF with NSAIDs has a strong effect on both *C. albicans* and *C. glabrata* biofilm production, while the synergistic effect of the combination is weak against *C. Guilliermondi* (Rosato et al., 2016). The *in vitro* interactions between aspirin, ibuprofen, and diclofenac sodium with commonly used azoles also have potential effects against planktonic and biofilm cells of *T. asahii* (Yang et al., 2016).

## Microsomal prostaglandin E synthase-1(mPGES-1)

PGES enzymes, which lie downstream of COXs, have been formed into several isoforms. Current evidence suggests that cytosolic PGES (cPGES) and microsomal prostaglandin E synthase-2 (mPGES-2) are constitutively expressed in cells and are coupled with COX-1 and COX-1/-2, respectively (Murakami et al., 2003). Microsomal PGES-1 (MPGES-1) is stimulus inducible and specifically couples with COX-2; it is the terminal enzyme in the biosynthesis of PGE_2_ and, ideally, does not affect the formation of other housekeeping PGs (Koeberle et al., 2010; Chandrasekhar et al., 2016). In Chandrasekhar's research, mPGES-1 exhibits selective inhibition of PGE_2_ in human epithelial cells, carcinoma cells (A549) and human whole blood treated with lip polysaccharides (LPS; Chandrasekhar et al., 2016). An mPGES-1 knock-out experiment shows that mPGES-1 deficient mice cannot induce the expression of mPGES-1 in response to LPS (Uematsu et al., 2002; Trebino et al., 2003). In various mice and rat inflammation models, mPGES-1 plays a pathogenic role in tumorigenesis (Fahmi, 2004), inflammation (Trebino et al., 2003; Iyer et al., 2009), and bone metabolism (Saha et al., 2005). An mPGES-1 inhibitor was found to be efficacious for arthralgia (Chandrasekhar et al., 2016) and is regarded as a promising influenza therapeutic target because of its ability to suppress the induction of pro-inflammatory genes (Park et al., 2016). In addition, mPGES-1 participated in various pathophysiological states in which both COX-1 and COX-2 are involved, implying that the role of the mPGES-1 enzyme is partially similar to that of COX (Murakami et al., 2002, 2003; Tanioka et al., 2003).

Non-selective COX inhibitors can result in many adverse effects, such as gastrointestinal complications and renal toxicity, mainly because COX inhibitors disturb the balance of anti-thrombotic prostacyclins (PGI_2_) and pro-thrombotic thromboxane A2 (TXA_2_) production (Bresalier et al., 2005). Though selective COX-2 inhibitors (coxibs) have an improved gastrointestinal tolerance, clinical studies have shown that COX-2 inhibitors lead to a small but significant increase in cardiovascular risk, which caused rofecoxib and valdecoxib to be withdrawn from the market (Buttgereit et al., 2001; Sun et al., 2007). MPGES-1, the terminal enzyme, is functionally linked to both COX-1 and COX-2 and can produce PGE_2_ with fewer side effects, and it can also maintain the TXA_2_ and PGI_2_ balance (Vidal et al., 2007) without influencing the 12-LOX and 15-LOX pathways (Martel-Pelletier et al., 2003). Currently, mPGES-1 inhibitors are emerging as the foremost agents in the treatment of inflammatory related diseases, but their antifungal activity is still not clear. Therefore, shifting focus to the use of more selective mPGES-1 inhibitors on the anti-fungal front as an alternative pharmacological approach may be a wise treatment strategy (Rådmark and Samuelsson, 2010). Several compounds, including MF-63, Triclosan and many natural products, are considered to be mPGES-1 inhibitors, such as Myrtucommulone from myrtle (Koeberle et al., 2009a), Arzanol from Helichrysum (Bauer et al., 2011), and Curcumin, which have anti-inflammatory and anti-carcinogenic properties (Koeberle et al., 2009b). These natural compounds can efficiently suppress mPGES-1 activity (IC50 = 0.3–10 μM) and reduce PGE_2_ levels (Korotkova and Jakobsson, 2014). In addition, licofelone as an mPGES-1 inhibitor has succeeded in reaching the required criteria in phase III clinical trials for treating osteoarthritis (Payandemehr et al., 2015). Pharmacodynamic studies in various animal models have confirmed the effectiveness of licofelone in many types of diseases, such as anti-asthmatic and anticonvulsant effects (Rotondo et al., 2002; Kulkarni and Singh, 2007; Payandemehr et al., 2015). Furthermore, licofelone was also identified as a class of dual mPGES-1/LOX inhibitors, and its ability to block both the mPGES-1 and 5-LOX pathways is considered superior to single interference.

## 5-lipoxygenase

The lipoxygenase pathway in human beings mainly consists of three enzymes, 5-, 12-, and 15-lipoxygenase (LOX). 5-LOX as a crucial enzyme in the arachidonic acid cascade can mediate 5-OH-eicosatetraenoic acid (5-HETE) and leucotrienes production with the assistance of 5-LO activating protein (FLAP). The human 5-LOX pathway is important for allergic diseases and inflammatory disorders (Murakami et al., 2002; Werz and Steinhilber, 2006). In the research of Mariana Morato-Marques, LTs and the 5-LO signaling pathway were shown to promote NADPH oxidase activation and ROI generation as well as enhance alveolar macrophage anti-fungal activity against *C. albicans*. The 5-LO-derived leukotrienes (LTs) secreted by alveolar macrophages (AMs) can eliminate *C. albicans* from the lungs (Morato-Marques et al., 2011). The general LOX inhibitor nordihydroguaiaretic acid (NDGA) does not inhibit PG production in mammalian systems; however, in Candida, NDGA demonstrated effective activity toward inhibition of PGE_2_ production in whole cells in a dose-dependent manner (Erb-Downward and Noverr, 2007). On the other hand, LOX inhibitor shows ability to block the production of PG by inhibiting PLA2 translocation to cellular membranes, and therefore interfering the level of AA release (Rossi et al., 2010; He et al., 2012). These evidences demonstrated that 5-LO can serve as a regulator of Candida infection, but the certain way of 5-lipoxygenase regulate Candida is not sure.

## Group IVA cytosolic phospholipase A2 (cPLA2α)

Cytosolic phospholipase A2α (cPLA2α) enzymes from membrane glycerophospholipids play a central role in regulating arachidonic acid release and PGs synthesis. The activity of CPLA2α is regulated by intracellular calcium mobilization and phosphorylation by mitogen-activated protein kinases (MAPKs). CPLA2α triggers PGE_2_ biosynthesis through COX-1 and can induce expression of *Il10, Ptgs2*, and *Nr4a2*, while suppressing *Tnf*α expression in MAPK by increasing cAMP (Yun et al., 2016). Other results suggest that in the Golgi apparatus, cPLA2, COX-2 and mPGES-1 can provide a beneficial system for PGE_2_ formation (Evans and Leslie, 2004; Yuan and Smith, 2015). In a cPLA2α knockout mouse experiment, the cPLA2α-deficient mice appeared to be severely arthritic, implying that cPLA2α is a key player in the pathogenesis of collagen (Hegen et al., 2003). The CPLA2α inhibitor arachidonyl trifluoromethyl ketone (ATK) plays an important role in ameliorating tissue injury and pain (Khan et al., 2015). The host cPLA2α can enhance *P. aeruginosa*-induced mouse mortality by mediating the 15-LOX and COX-2 signaling pathways (Guillemot et al., 2014). In short-term infection of *C. albicans*, macrophages activate cPLA2α to preferentially initiate arachidonic acid release for eicosanoid production, and cPLAα is mediated by the b-Glucan Receptor Dectin-1, which can promote *C. albicans* to stimulate cPLAα release of AA and contribute to generating the signals to activate cPLA2α (Suram et al., 2006; Parti et al., 2010). In brief, *C. albicans* engage with multiple receptors on macrophages to provide signals to activate cPLA2α and produce eicosanoids, which are keys for modulating inflammation and fungal-related disease (Suram et al., 2010). Therefore, inhibiting cPLA2α may be a solution to treat *C. albicans* infection by reducing PGE_2_ production.

## Others

Except for the enzymes discussed above, there are many other factors that regulate enzyme activity and PGE_2_ biosynthesis. Under hypoxia, intricate temperature-CO2 modulation is able to reduce hyphal formation of *C. albicans* and influence fungal virulence (Lu et al., 2013; Desai et al., 2015). This may be because PGE_2_ production was decreased while prostacyclin and thromboxane production were increased in hypoxia (Blumenstein et al., 2001). When ROS or ROS-generating xenobiotics are present at low levels, they can activate the prostanoid synthesis pathway and simultaneously activate cPLA2, COX2, and mPGES-1 (Korbecki et al., 2013). Marnett showed that in a model of NOS deficient mice, PGE_2_ production in macrophages was significantly reduced and that in urine, the level of PGE_2_ in the NOS deficient groups was decreased by 78% compared to the control groups (Marnett et al., 2000). In Devaux's study, the results suggest that NOS2-derived NO can activate the mPGES-1 pathway when COX-2 protein expression is absent and that inhibiting the activity of NOS2 decreases the concentrations of PGE_2_ induced by LPS (Devaux et al., 2001). Hence, NOS2 inhibitors are useful to inhibit the production of PGE_2_ via COX-2-independent mechanisms. Hemeoxygenases (HOs) have been shown to regulate the levels of eicosanoids derived in the cyclooxygenase, lipoxygenase, and cytochrome P450 monooxygenase (CYP) pathways. Up regulation of HO-1 or increased HO activity can suppress PGE_2_, inducing production of 15-hydroxyeicosatetraenoic acid (HETE), lipoxins, and resolvins (Abraham and Kappas, 2008; Fox et al., 2013). In mammalian cells, sciadonic acid cannot be directly metabolized to PGE_2_, but it can compete with AA for incorporation into phospholipids and lead to a reduction in PGE_2_ production (Ells et al., 2012). Several factors influencing PGE_2_ production were summarized and evidence showed that they also impact the growth of Candida.

## Conclusion

The arachidonic acid cascade plays a central role in fungal morphogenesis, yeast-hypha conversion, and biofilm formation. In *C. albicans* cells, PGE_2_ as a virulence factor can regulate fungal growth, colonize, and survive during infection (Noverr and Huffnagle, 2004). PGE_2_ also plays an important role in promoting the formation of Candida biofilms. Some studies have shown that the less PGE_2_ production might contribute to the lower level of fungal virulence (Ells et al., 2011). Therefore, an approach to overcome the virulence of PGE_2_ is sorely needed. A series of enzymes that are involved in the synthesis of PGE_2_ described above have been studied widely. During *in vitro* and *in vivo* studies, COX inhibitors, such as indomethacin, ibuprofen and aspirin, have been confirmed to have antifungal ability by suppressing *C. albicans* PGE_2_ production and biofilm formation (Liu et al., 2014). The antifungal activity of COX inhibitors is weak in both planktonic and biofilm cells, but in a combined therapy, aspirin can increase fluconazole susceptibly to *C. albicans*. In addition, the possible antifungal activity of mPGES-1 inhibitors against *C. albicans* was also discussed. As the terminal enzyme downstream of COX-2, mPGES-1 can catalyze the biosynthesis of PGE_2_ with fewer side effects and, ideally, cannot affect the formation of other housekeeping PGs. Licofelone, as an mPGES-1 inhibitor, has succeeded in reaching the required criteria in phase III clinical trials for treating osteoarthritis. Furthermore, discovery of the possible antifungal ability of mPGES-1 is exceedingly promising. Other factors that influence PGE_2_ production, such as ROS, NOS, and HOs, are also mentioned in this review.

Hence, further academic research is needed to provide new insights that are able to further our understanding of the importance and role of PGE_2_ in *C. albicans* and the mechanism of the potential antifungal agents related to the arachidonic acid cascade, as well as to developing an approach to discover new antifungal drugs for resistant *C. albicans*.

## Author contributions

**Figure 1:**
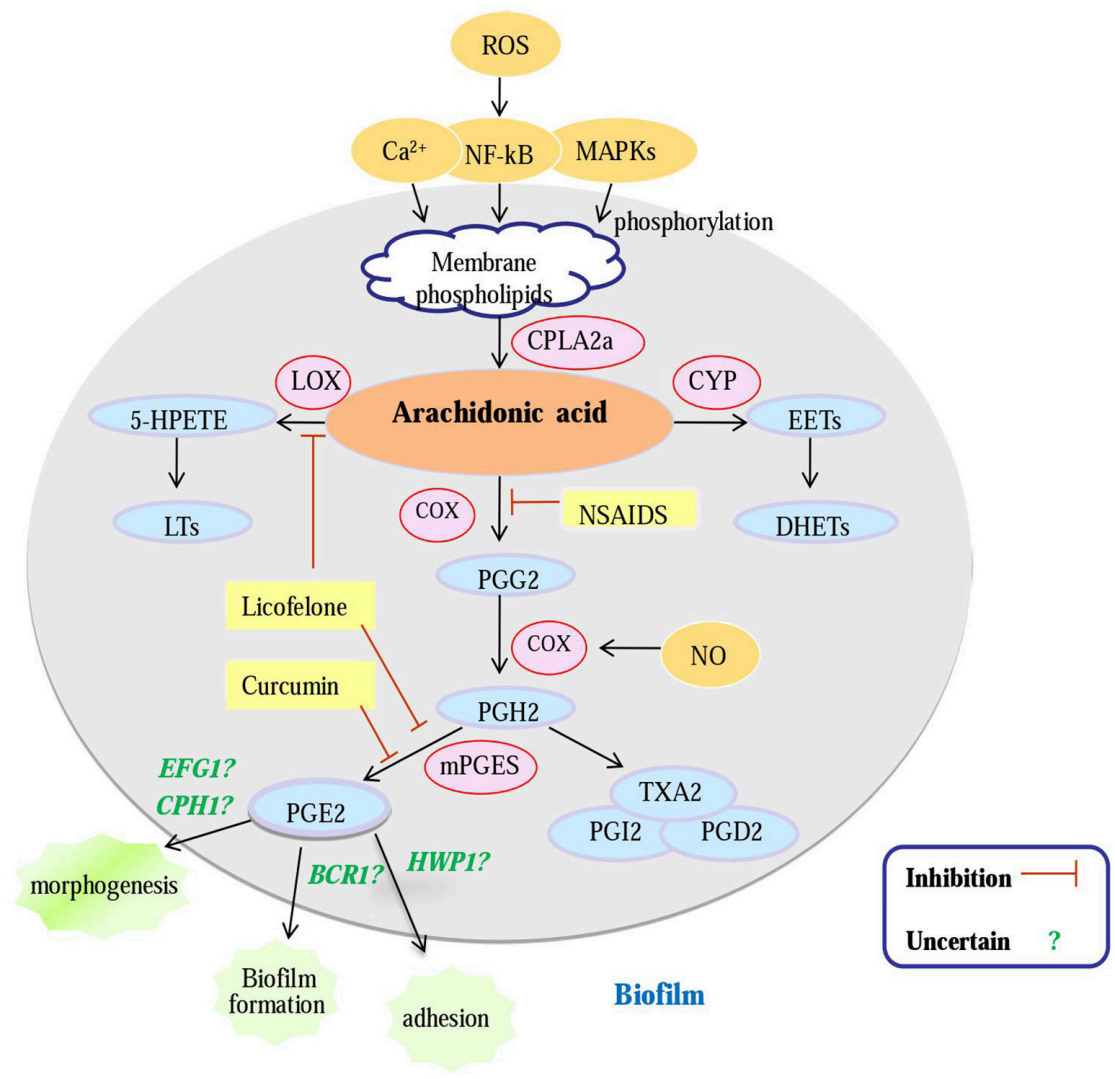
**An overview of potential antifungal targets against a Candida biofilm based on an enzyme in the arachidonic acid cascade**. Gene (green); enzyme (pink); metabolites (blue); inhibitors (yellow); Question mark: PG E2 can regulate biofilm morphogenesis, biofilm formation and biofilm adherence, whether PGE2 modulates biofilm formation of *C. albicans* by regulating these genes is uncertain; cPLA2a, cytosolic PLA2a; CYP, cytochrome P450; LOX, lipoxygenase; COX, cyclooxygenase; mPGES, microsomal; prostaglandin E2 synthase; NSAIDS, Non-Steroidal Antiinflammatory Drugs; PGG2, prostaglandin G2; PGH2, prostaglandin H2; PGE2, prostaglandin E2; NO, nitric oxide.

XL wrote the review and created the Figure 1, SS, DW, CY, TL, JL helped with it.

### Conflict of interest statement

The authors declare that the research was conducted in the absence of any commercial or financial relationships that could be construed as a potential conflict of interest.
